# Exercise ameliorates high fat diet induced cardiac dysfunction by increasing interleukin 10

**DOI:** 10.3389/fphys.2015.00124

**Published:** 2015-04-22

**Authors:** Varun Kesherwani, Vishalakshi Chavali, Bryan T. Hackfort, Suresh C. Tyagi, Paras K. Mishra

**Affiliations:** ^1^Department of Cellular and Integrative Physiology, University of Nebraska Medical CenterOmaha, NE, USA; ^2^Department of Physiology and Biophysics, University of LouisvilleLouisville, KY, USA; ^3^Department of Anesthesiology, University of Nebraska Medical CenterOmaha, NE, USA

**Keywords:** obesity, heart failure, inflammation, interleukin 10, TNF-α, echocardiography, Masson Trichrome

## Abstract

Increasing evidence suggests that a sedentary lifestyle and a high fat diet (HFD) leads to cardiomyopathy. Moderate exercise ameliorates cardiac dysfunction, however underlying molecular mechanisms are poorly understood. Increased inflammation due to induction of pro-inflammatory cytokine such as tumor necrosis factor-alpha (TNF-α) and attenuation of anti-inflammatory cytokine such as interleukin 10 (IL-10) contributes to cardiac dysfunction in obese and diabetics. We hypothesized that exercise training ameliorates HFD- induced cardiac dysfunction by mitigating obesity and inflammation through upregulation of IL-10 and downregulation of TNF-α. To test this hypothesis, 8 week old, female C57BL/6J mice were fed with HFD and exercised (swimming 1 h/day for 5 days/week for 8 weeks). The four treatment groups: normal diet (ND), HFD, HFD + exercise (HFD + Ex) and ND + Ex were analyzed for mean body weight, blood glucose level, TNF-α, IL-10, cardiac fibrosis by Masson Trichrome, and cardiac dysfunction by echocardiography. Mean body weights were increased in HFD but comparatively less in HFD + Ex. The level of TNF-α was elevated and IL-10 was downregulated in HFD but ameliorated in HFD + Ex. Cardiac fibrosis increased in HFD and was attenuated by exercise in the HFD + Ex group. The percentage ejection fraction and fractional shortening were decreased in HFD but comparatively increased in HFD + Ex. There was no difference between ND and ND + Ex for the above parameters except an increase in IL-10 level following exercise. Based on these results, we conclude that exercise mitigates HFD- induced cardiomyopathy by decreasing obesity, inducing IL-10, and reducing TNF-α in mice.

## Introduction

Sedentary lifestyle and high fat diet (HFD) are major risk factors for diabetes and heart failure (Lopez et al., 2006; Pedersen and Febbraio, 2012; Benatti and Pedersen, 2015). HFD in association with sedentary lifestyle increases the probability of obesity. Obesity accentuates systemic inflammation (Lumeng and Saltiel, 2011). Inflammation is associated with increased levels of pro- inflammatory cytokines such as tumor necrosis factor alpha (TNF-α), and decreased levels of anti-inflammatory cytokines such as interleukin 10 (IL-10). It is demonstrated that TNF-α is produced in the heart by myocardial macrophages and cardiac myocytes and contributes to myocardial dysfunction in an autocrine manner (Meldrum, 1998). TNF-α promotes pathological cardiac remodeling by inducing hypertrophy and fibrosis in the heart (Sun et al., 2007). Because of the increasing role of TNF-α in the heart disease, TNF-α antagonists were used in clinical trials for therapy for heart failure, however they had conflicting results and were not successful (Gupta and Tripathi, 2005). TNF-α-mediated inflammation is balanced by secretion of the anti-inflammatory cytokine IL-10, and low IL-10/TNF-α are associated with pathophysiology of heart failure (Dopheide et al., 2015). IL-10 has pleiotropic effect on regulation of inflammation (Moore et al., 1993; Wang et al., 1994b) and low levels of IL-10 is used as a predictor of heart failure (Kirkpantur et al., 2008; Parissis et al., 2009). It is reported that enhanced levels of IL-10 by Atorvastatin ameliorates cardiac dysfunction in myocardial infarction (Stumpf et al., 2009). Therefore, inhibition of TNF-α and upregulation of IL-10 is protective to the heart. Although IL-10 prevents HFD-induced insulin resistance, the effect of HFD on TNF-α and IL-10 in the heart is unclear.

Exercise has many beneficial effects on cardiac function (Petersen and Pedersen, 2005). Empirical studies and clinical investigations demonstrate that endurance exercise improves cardiac function in the failing heart (Gielen et al., 2015; Zilinski et al., 2015). Swimming has many beneficial effects on the heart including a decrease in pro-inflammatory markers (Geenen et al., 1988; Cechella et al., 2014). Furthermore, exercise has been shown to increase anti-inflammatory cytokines including IL-10 (Steensberg et al., 2003; Pedersen et al., 2007; Viana et al., 2014; Benatti and Pedersen, 2015). IL-10 is involved in the protection against HFD, LPS-induced inflammation, and hyper-insulinemia (Grant et al., 2014). However, the molecular mechanism by which aerobic exercise ameliorates cardiac dysfunction is poorly understood. Here, we investigated the role of exercise training in mitigation of HFD-induced inflammation, pathological remodeling, and cardiac dysfunction in female mice.

## Materials and methods

### Animals

Eight week old, female C57BL/6J mice were procured from Jackson Laboratory (Bar Harbor, ME, USA). Animals were housed with controlled temperature (22–24°C) and 12 h light/dark cycle. Mice were allowed free access to food and drinking water. All animal procedures were reviewed and approved by the Institutional Animal Care and Use Committee (IACUC) of the University of Nebraska Medical Center and performed under the guidelines of the National Institute of Health (NIH). Most of the studies related to diet and exercise are skewed toward males therefore to bridge this gap, only female mice were used in our study.

### Exercise and HFD

To study the effect of HFD mediated cardiac dysfunction, we fed HFD (HarlanTD.08811) or normal diet (ND) to female mice for 8 weeks. The HFD contained 46.1% fat, while ND had 9.8% fat components. HFD has been shown to reduce glucose tolerance and increase insulin resistance mimicking type 2 diabetes (Winzell and Ahren, 2004). To assess the effect of exercise on HFD mediated cardiac dysfunction, both ND and HFD mice were exposed to swimming exercise 1 h/day for 5 days/week for a total of 8 weeks. Temperature of the water was kept ambient (31 ± 1°C). For experiment design, we selected four groups of mice: ND, HFD, ND with exercise training (ND + Ex), and HFD with exercise training HFD + Ex.

### Measurement of LV function by echocardiography

Details of this protocol has been described elsewhere (Mishra et al., 2011). Briefly, mice were anesthetized by isoflurane. The M-Mode echocardiography was performed with Vevo2100 imaging system by positioning the transducer on the hemi-thorax region of the mouse. Fractional shortening (FS) was calculated from the formula: %FS = 100 × (LVIDd—LVIDs)/LVIDd. LVIDd stands for left ventricular internal diameter in diastole, and LVIDs denotes left ventricular internal diameter in systole (Mishra et al., 2011). Echocardiography was performed in the laboratory of Dr. Suresh Tyagi at the University of Louisville.

### Western blotting

Heart proteins were extracted using RIPA lysis buffer. Total protein was quantified by Bradford assay and the expression of specific proteins was determined by Western blotting as described previously (Chavali et al., 2014). The primary antibodies for TNF-α (Cell Signaling, 3707s), IL-10 (EMD Millipore, ABF13), and β-actin (Santacruz Biotech, sc-47778) were diluted 1:1000 and blots were incubated in primary antibody overnight at 4°C. HRP-linked secondary antibodies against rabbit (Santacruz Biotech, sc-2054) or mouse (Santacruz Biotech, sc-2005) were used in 1:4000 dilution. Blots were incubated in secondary antibodies for 1 h at room temperature (RT). The membranes were exposed with HRP substrate and imaged with BioRad ChemiDoc. Densitometry analyses was performed by ChemiDoc software (BioRad, CA, USA).

### Immunohistochemistry

Frozen heart tissue from different groups were cryosectioned (5 μm) using Cryostar NX50 (Thermo Scientific). Sections were fixed in 4% paraformaldehyde for 30 min and made permeable using 0.5% Triton X-100 in PBS for 30 min. Sections were then blocked in 1% BSA followed by overnight incubation in anti-TNF-α (1:500) and anti-IL-10 (1:500) antibody at 4°C. The sections were washed with PBS three times for 5 min and incubated with anti-rabbit Alexa Fluor 594 (Life Technologies, A11012) for 1 h at RT. Subsequently, sections were washed, stained with the nuclear stain DAPI and imaged with fluorescence microscope (EVOS, Life technologies).

### Masson trichrome staining

To observe fibrosis in tissue sections of different groups, Masson Trichrome staining was performed following company's protocol (Thermo Scientific cat. No. 87019). In brief, sections were placed in Bouin's fluid at 56°C for 1 h. Sections were rinsed in deionized water and stained with Biebrich scarlet-acid fuchsin for 10 min. Sections were then placed in phosphotungstic-phosphomolybdic acid solution for 15 min followed by aniline blue staining for 30 min. Sections were placed in 1% acetic acid solution for 1 min, rinsed, dehydrated, mounted, and imaged under microscope (Mishra et al., 2012).

### Statistical analysis

One–Way of analyses of variance (ANOVA) was used for finding the difference of means among the four groups. Student *t*-test was performed to calculate the significant differences in means between the two groups. *P* < 0.05 was considered statistically significant. Color intensity of the microscopic images was measured by Image J software.

## Results

### HFD increases mean body weight and blood glucose levels

**Figure 1:**
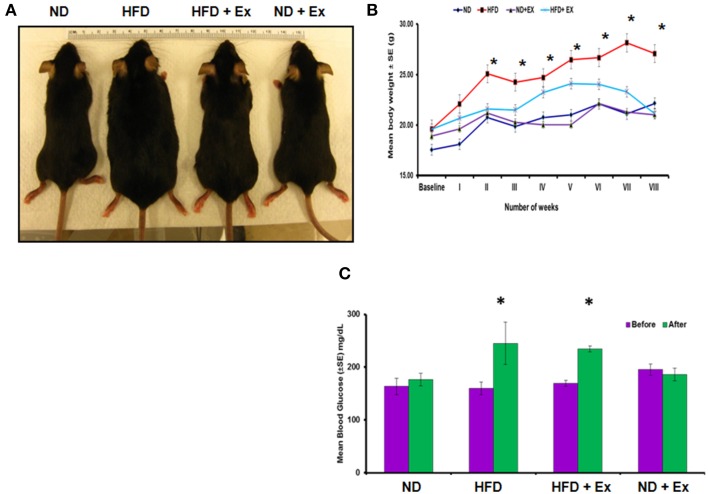
**High fat diet (HFD) induces obesity in female mice**. **(A)** Photographs of representative mice of each group (ND, HFD, ND + Ex, HFD + Ex) after 8 weeks. **(B)** Mean body weight in each group over a period of 8 weeks. **(C)** Blood glucose levels in each group before and after 8 weeks of HFD. All the data have been represented as mean ± SE. ^*^P < 0.05 compared to ND.

To validate HFD- induced obesity, C57BL/6J mice were fed a HFD. Physical appearance of mice, body weights, and random blood glucose levels were measured to confirm the HFD induced an obese phenotype (Figure 1). Mice fed with HFD showed a significant increase in mean body weight as compared to other groups (*P* < 0.05), whereas exercise maintained the body weight in the HFD + Ex group similar to the group fed with ND (Figure 1B). Although exercise is reported to reduce blood glucose level (Correa et al., 2015), we did not find significant decrease in the level of blood glucose in HFD + Ex group (Figure 1C). It could be due to less duration and/or intensity of exercise.

### Exercise mitigates inflammation in mice fed with HFD

**Figure 2:**
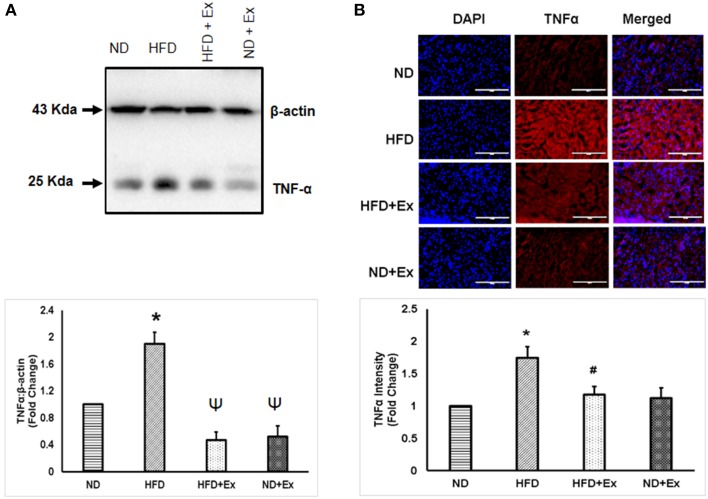
**(A)** Expression of TNF-α protein levels in different groups. β-actin was used as a loading control. Bar diagram shows fold changes (*N* = 6) in TNF-α expression. **(B)** Microscopic images of tissue sections of different groups stained with rabbit anti-TNF antibody and anti-rabbit alexa flour 594. Bar diagram shows the fold changes (*N* = 6) in TNF-α expression relative to normal diet. Values are represented as a mean ± SE (*N* = 5–6). ^*^P < 0.05 compared to ND; ^Ψ^*P* < 0.05 compared ND and HFD; ^#^*P* < 0.05 compared HFD.

**Figure 3:**
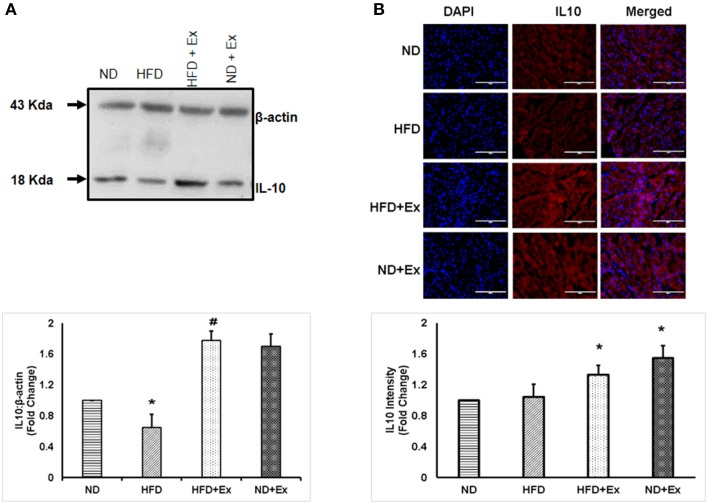
**(A)** Expression of IL-10 protein levels in different groups of animals. β - actin was used as a loading control. Bar diagram shows fold change (*N* = 4) in IL-10 expression relative to normal diet. **(B)** Microscopic images of tissue sections of different groups stained with rabbit anti-IL-10 antibody and anti-rabbit alexa flour 594. Bar diagram shows fold changes in IL-10 expression relative to normal diet. Values are represented as a mean ± SE (*N* = 4–5). ^*^P < 0.05 compared to ND; ^#^*P* < 0.05 compared HFD.

To determine the role of HFD inducing inflammation, the expression of TNF-α and IL-10 was measured in heart tissue. The levels of TNF-α and IL-10 were different among the groups. HFD increased levels of TNF-α, a known hallmark of diabetic inflammation, in cardiac tissue, which was alleviated by exercise (Figure 2). The anti-inflammatory cytokine, IL-10 was found to be upregulated in both exercise groups compared to sedentary controls (Figure 3). These data suggest that HFD increases inflammation, whereas exercise mitigates inflammation by inducing anti-inflammatory IL-10 in cardiac tissue.

### Exercise mitigates HFD- induced cardiac fibrosis and cardiac dysfunction

**Figure 4:**
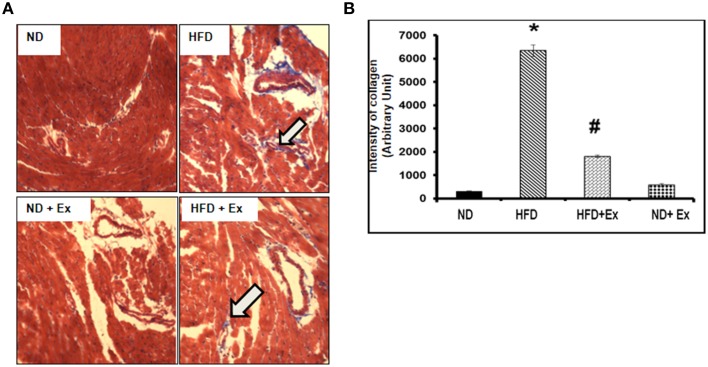
**(A)** Masson Trichrome staining assessing fibrosis in heart sections of each group. Arrows denote examples of fibrosis (blue stain). **(B)** Bar diagrams represent the quantification of fibrotic collagen (blue) content of image. Data represents mean ± SE (*N* = 4). ^*^P < 0.05 compared to ND, and ^#^*P* < 0.05 compared to HFD.

**Figure 5:**
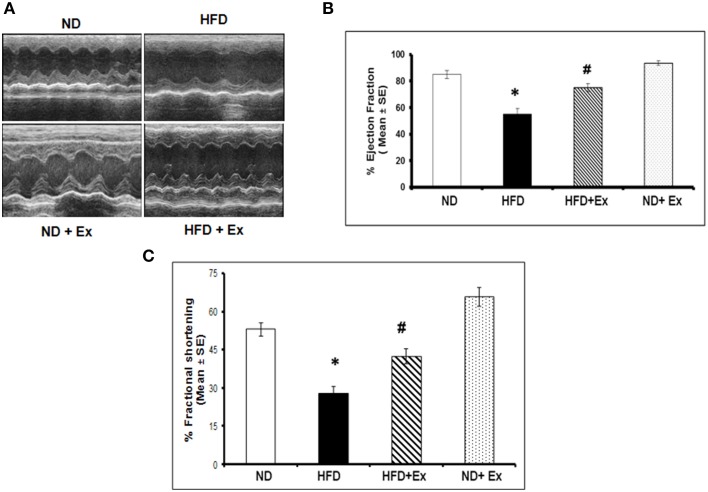
**(A)** Representative M-mode echocardiography after 8 weeks of treatment. **(B)** The bar graphs represent percentage of ejection fraction (%EF) and **(C)** percentage fractional shortening (%FS). Data represents mean ± SE (*N* = 4). ^*^P < 0.05 compared to ND, and ^#^*P* < 0.05 compared to HFD.

Masson Trichrome staining and echocardiography were done to assess the effect of HFD on cardiac tissue fibrosis and cardiac dysfunction. Mice fed with HFD showed significant fibrosis in perivascular as well as interstitial regions in heart tissue (*P* < 0.05) (Figure 4). Increased fibrosis in extracellular tissues impairs the cardiac function and leads to cardiomyopathy (Huynh et al., 2014). HFD + Ex mice showed decreased fibrosis and improved cardiac function compared to HFD (Figures 4, 5) suggesting that exercise mitigates HFD-mediated pathological cardiac remodeling. Echocardiogram results showed significant decrease in the %FS and %EF in HFD (Figure 5). However, exercise prevented the HFD induced decrease in %EF and %FS in HFD + Ex mice. There was no significant difference in cardiac function as measured by %EF and %FS in the ND and HFD + Ex (Figure 5). These data suggest that exercise mitigates HFD-mediated myocardial fibrosis and ameliorates cardiac dysfunction.

## Discussion

**Figure 6:**
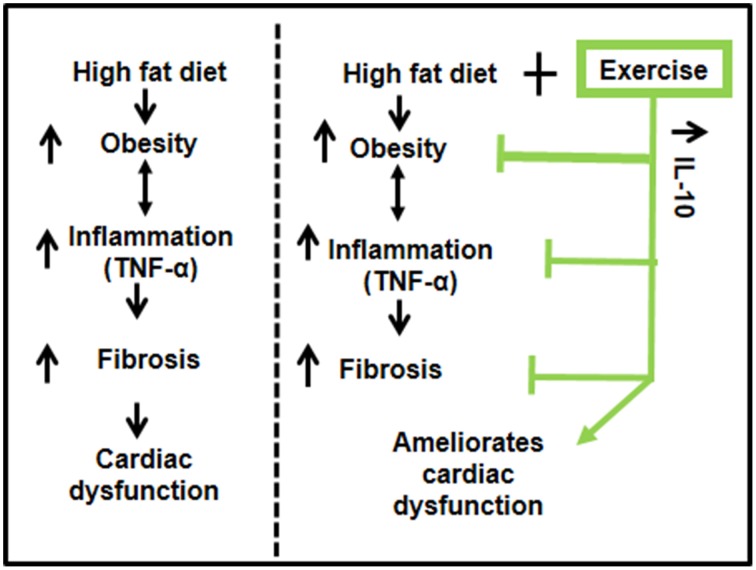
**A schematic representation of the cardioprotective role of exercise on high fat diet mediated cardiac dysfunction**. High fat diet leads to obesity, which enhances the systemic inflammation (TNF-α) and causes fibrosis leading to cardiac dysfunction. Exercise decreases high fat diet induced obesity, upregulates anti-inflammatory IL-10, which reduces inflammation, mitigates fibrosis, and ameliorates cardiac dysfunction.

Our results suggest that exercise ameliorates HFD-mediated cardiac dysfunction in female mice. HFD induced obesity and inflammation (TNF-α) resulting in fibrosis and decreased percentage ejection fraction. Exercise increased levels of IL-10 in cardiac tissue, reduced the pro-inflammatory TNF-α, mitigated fibrosis and ameliorated cardiac dysfunction (Figure 6). Our results support previous literature that exercise has beneficial effects in healthy, as well as in pathological conditions such as diabetes and obesity (DeBlieux et al., 1993; Broderick et al., 2005; Petersen and Pedersen, 2005; Mishra et al., 2011).

Obesity is a risk factor for type 2 diabetes, therefore a HFD was used to simulate an obese condition (Schilling and Mann, 2012). Consistent with a previous study, our data show that the HFD increased blood glucose levels and mean body weights, suggesting a diabetic phenotype was induced (Winzell and Ahren, 2004). In the diabetic condition, high blood glucose and free fatty acids induce inflammation in cardiac tissues (Schilling and Mann, 2012). The level of TNF-α is increased whereas that of IL-10 is decreased in the diabetic heart (Chavali et al., 2013). Excessive TNF-α leads to increased fibrosis and apoptosis of cardiomyocytes (Sun et al., 2007; Chiong et al., 2011). We observed an increased level of TNF-α in HFD group compared to ND group (Figure 2). TNF-α is pro-inflammatory and causes a risk lipid profile by increasing the level of triglycerides and LDL (Popa et al., 2005), suggesting that TNF-α is associated with obesity. TNF-α induces the expression of matrix metalloproteinase, which is involved in activation of TGFβ1 secreted by myocytes and macrophages (Sun et al., 2004). TGFβ1 increases synthesis of collagen I and III and deregulates collagen turnover. Increased collagen changes the stiffness of ventricle walls and interferes with normal cardiac function, resulting in increased fibrosis and heart failure (Searls et al., 2004). Exercise training attenuated cardiac fibrosis (HFD + Ex) that was induced by the HFD (Figure 4) consistent with an earlier study that found exercise reduced fibrosis by keeping inflammatory stress low (Searls et al., 2004).

During exercise, muscles release myokines that are involved in tissue growth, repair, and anti-inflammatory responses (Petersen and Pedersen, 2005; Benatti and Pedersen, 2015). IL-6 is the primary myokine released in response to exercise and has been shown to increase levels of the anti-inflammatory IL-10 and decrease levels of the pro-inflammatory TNF-α (Steensberg et al., 2003; Petersen and Pedersen, 2005; Benatti and Pedersen, 2015). IL-10 has been shown to mitigate cardiac dysfunction by decreasing cardiac fibrosis (Petersen and Pedersen, 2005). The HFD decreased cardiac IL-10 protein levels compared to ND controls suggesting IL-10 as an important cytokine for mediating fibrosis. Exercise training significantly increased levels of IL-10 in cardiac tissues (Figure 3) and previous reports show IL-10 inhibits TNF-α (Wang et al., 1994a,b; Pretolani, 1999).

In conclusion, swimming exercise reversed the pathological remodeling induced by a HFD by increasing cardiac IL-10 levels and decreasing TNF-α levels. Shifting the cytokine profile from pro-inflammatory (TNF-α) to anti-inflammatory (IL-10) measured in cardiac tissue may be the link in attenuating fibrosis. Further, exercise mitigated the decline in the % FS and % EF induced by the HFD, confirming the cardioprotective role of exercise.

## Future direction

Our results show that exercise induces IL-10 and mitigates HFD-induced cardiac dysfunction in mice. Future studies on administration of IL-10 in HFD fed mice will reveal the cardioprotective effect of IL-10 in HFD-mediated cardiac dysfunction.

### Conflict of interest statement

The authors declare that the research was conducted in the absence of any commercial or financial relationships that could be construed as a potential conflict of interest.

## Abbreviations

TNFα: Tumor necrosis factor alpha; IL-10, Interleukin 10; HFD, High fat diet; ND, Normal diet; Ex, Exercise
DCM: Diabetic cardiomyopathy.
